# Unlikely Double Trouble: Two Cases of Haemobilia From One Motor Vehicle Accident

**DOI:** 10.7759/cureus.76491

**Published:** 2024-12-27

**Authors:** Supun M Bakmiwewa, Salah Ayoubi, Mina Sarofim, Katherine Gibson

**Affiliations:** 1 Department of Colorectal Surgery, Liverpool Hospital, Sydney, AUS; 2 Faculty of Medicine, University of New South Wales, Sydney, AUS; 3 Faculty of Medicine, University of Sydney, Sydney, AUS

**Keywords:** blunt abdominal trauma, cholecystectomy, contusion, gallbladder injuries, motor vehicle

## Abstract

Blunt abdominal trauma frequently results in visceral injury to either solid or hollow organs; however, injury to the gallbladder is rare. This is most likely due to the anatomical position of the gallbladder, which is well-insulated posterior to the liver and rib cage. Gallbladder injuries can be in the form of avulsion, contusion, or laceration. In the same clinical spectrum as contusion is haemobilia, which is defined as bleeding into the biliary system and may result from injury to any of the structures associated with the biliary tree (liver, gallbladder, bile ducts or pancreas). Risk factors for traumatic injury to the gallbladder include distension of the gallbladder due to recent alcohol ingestion or fasting and deceleration-type mechanisms, which may cause compression of the gallbladder against the spine. Haemobilia is an important diagnosis with important clinical implications. If undiagnosed, traumatic haemobilia can result in complications such as delayed gallbladder perforation secondary to local ischemia or cholecystitis secondary to obstructing blood clots, which can add to the associated injury burden in trauma patients. Although extremely rare, we present an unlikely scenario of two patients who were involved in the same motor vehicle crash, and both were found to have traumatic haemobilia that required surgical management with cholecystectomy. A high degree of suspicion is required to avoid missed/delayed diagnosis of haemobilia, which may have serious complications that can lead to increased morbidity and mortality in trauma patients.

## Introduction

Blunt abdominal trauma, commonly caused by motor vehicle accidents, sports injuries, falls and physical assaults, frequently results in visceral injury to either solid or hollow organs [1]. Signs and symptoms of blunt abdominal trauma can include pain, distension, bruising, and rectal bleeding, and the evaluation method largely depends on the haemodynamic stability of the patient and includes extended focused assessment with sonography for trauma (eFAST), computed tomography (CT) imaging and trauma operations [2]. Injury to the gallbladder, however, is exquisitely rare and is reported in ~2% of all abdominal traumas [3]. It can be in the form of avulsion, contusion or laceration, and these can lead to further complications such as delayed gallbladder perforation and gallstone formation due to clots or cholecystitis [4]. In the same clinical spectrum as contusion is haemobilia, which is defined as blood within the biliary tree [3,5]. We present an unlikely scenario of two patients who were involved in the same motor vehicle crash (MVC), and both were found to have traumatic haemobilia that required surgical management with cholecystectomy.

## Case presentation

A 50-year-old female driver and a 61-year-old male passenger were involved in a 90 km/hour MVC versus a tree. Both were restrained with seatbelts and airbags deployed on impact.

**Figure 1:**
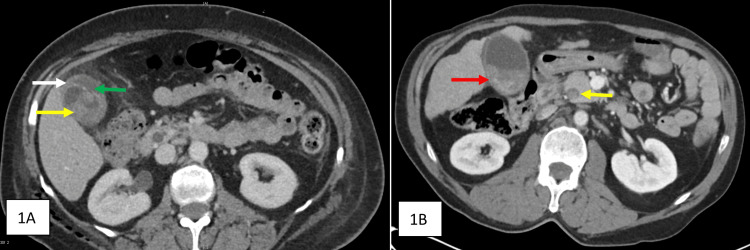
CT scans of the two patients during admission 1A: CT mesenteric angiogram (CTMA) for the female driver at 72 hours post-injury. Hyper-dense material within gallbladder lumen (yellow arrow), discontinuity of gallbladder wall (green arrow) and pericholecystic fluid (white arrow) are seen, which indicate a traumatic perforation with haemobilia. 1B: Initial CT of abdomen and pelvis for the male passenger at the time of presentation. Hyper-density in the dependent portion of the gallbladder lumen (red arrow) and mild pericholecystic fluid can be seen. The CBD is dilated and also contains hyperdense material (yellow arrow).

The driver's CT scan revealed splenic contusions and mild pericholecystic free fluid. Progress CT mesenteric angiogram (CTMA) at 72 hours was performed for ongoing abdominal pain and tenderness, which demonstrated discontinuity in the anterior gallbladder wall, new hyperdense material in the gallbladder, and a moderately dilated common bile duct (CBD). This was consistent with traumatic haemobilia and gallbladder perforation (Figure 1A), and invasive angiography did not demonstrate any active haemorrhage. She underwent a laparoscopic cholecystectomy, which confirmed a contained perforation at the gallbladder fundus. An intra-operative cholangiogram showed a filling defect in the CBD consistent with a clot, which was successfully flushed. Histopathology showed a thick-walled gallbladder with a focally haemorrhagic serosal surface.

The passenger's CT scan at presentation showed a segment VII liver laceration and a layering hyper-density within a dilated CBD with mild pericholecystic free fluid (Figure 1B). Progress CTMA again performed for ongoing abdominal pain at 72 hours showed worsening oedema, pericholecystic fluid and increased hyperdense material within the gallbladder lumen. These findings were consistent with traumatic haemobilia. This patient also underwent a laparoscopic cholecystectomy, which similarly confirmed a gallbladder perforation, but in this instance, at the Hartmann's pouch. Histopathology findings were consistent with haemorrhage, fibrosis and formation of granulation tissue within the gallbladder wall.

Both patients had an uneventful recovery from surgery and were subsequently discharged.

## Discussion

Blunt abdominal trauma can result in organ injury in up to one-fifth of cases [3]. Gallbladder injury secondary to trauma is rare, reportedly as low as 1-3% of patients undergoing trauma laparotomy, and literature is limited to case reports [5]. This is most likely due to the anatomical position of the gallbladder, which is well-insulated posterior to the liver and rib cage. As a corollary of this, gallbladder injury is most likely to be associated with other intra-abdominal injuries [5]. Risk factors for injury to the gallbladder include distension of the gallbladder due to recent alcohol ingestion or fasting and deceleration-type mechanisms, which may cause compression of the gallbladder against the spine [5].

Gallbladder injury can be classified as contusion/haemobilia, avulsion or laceration/perforation [3]. Haemobilia is defined as bleeding into the biliary system and may result from injury to any of the structures associated with the biliary tree, such as the liver, gallbladder, bile ducts or pancreas, and can be a life-threatening pathology [6,7]. If undiagnosed, traumatic haemobilia can result in complications such as delayed gallbladder perforation secondary to local ischemia or cholecystitis secondary to obstructing blood clots, which can add to the associated injury burden and increase the risk of morbidity and mortality in trauma patients.

Management of the isolated gallbladder injury is dependent on the extent of the injury. Simple contusion or partial avulsion may be treated conservatively [8,9]. Invasive angiography may be useful in controlling associated haemorrhage [7]. More severe injuries require treatment with cholecystectomy, and both open or laparoscopic approaches may be considered depending on the surgeon's ability and haemodynamic stability [3]. Both cases presented here had established complications of haemobilia in the form of gallbladder perforation that necessitated surgical management later during their admission.

Confirming the diagnosis of traumatic gallbladder injury/haemobilia can be difficult, and most injuries are found at the time of trauma operations [8]. CT imaging can be useful in identifying gallbladder pathology as well as its complications (with a reported CT specificity and sensitivity for blunt abdominal injuries of 94-100% and 96-100%, respectively) and pre-operative CT findings that suggest this are pericholecystic fluid, ill-defined contours of the gallbladder wall, collapsed gallbladder lumen and intraluminal haemorrhage [10-12]. The two cases presented here demonstrate the difficulty of pre-operative diagnosis of haemobilia in trauma patients based on initial imaging and highlight the importance of progress imaging in the diagnosis of haemobilia, particularly if there are ongoing symptoms in otherwise hemodynamically stable patients. A high degree of suspicion should be placed during the period of observation to avoid complications of a missed/delayed diagnosis of haemobilia.

## Conclusions

Traumatic gallbladder injury with haemobilia is outstandingly rare and represents only a small portion of all abdominal trauma injuries. CT imaging can be helpful in identifying gallbladder injuries and its complications. Though rare, we describe here a case of synchronous traumatic haemobilia from a single MVC, which is the first such case in literature to our knowledge. It is important to have a high index of suspicion, particularly in the intoxicated or fasted patient involved in a deceleration type event, to avoid missed/delayed diagnosis of haemobilia, which may have serious complications that can lead to increased morbidity and mortality in trauma patients.

## References

[1] Nishijima DK, Simel DL, Wisner DH, Holmes JF (2012). Does this adult patient have a blunt intra-abdominal injury?. JAMA.

[2] O'Rourke MC, Landis R, Burns B (2023). Blunt abdominal trauma. StatPearls [Internet].

[3] Jaggard MK, Johal NS, Choudhry M (2011). Blunt abdominal trauma resulting in gallbladder injury: a review with emphasis on pediatrics. J Trauma.

[4] Jang H, Park CH, Park Y, Jeong E, Lee N, Kim J, Jo Y (2021). Spontaneous resolution of gallbladder hematoma in blunt traumatic injury: a case report. World J Clin Cases.

[5] Tudyka V, Toebosch S, Zuidema W (2007). Isolated gallbladder injury after blunt abdominal trauma: a case report and review. Eur J Trauma Emerg Surg.

[6] Cathcart S, Birk JW, Tadros M, Schuster M (2017). Hemobilia: an uncommon but notable cause of upper gastrointestinal bleeding. J Clin Gastroenterol.

[7] Forlee M, Krige J, Welman C, Beningfield S (2004). Haemobilia after penetrating and blunt liver injury: treatment with selective hepatic artery embolisation. Injury, Int J Care Injured.

[8] Soderstrom CA, Maekawa K, DuPriest RW Jr, Cowley RA (1981). Gallbladder injuries resulting from blunt abdominal trauma: an experience and review. Ann Surg.

[9] Birn J, Jung M, Dearing M (2012). Isolated gallbladder injury in a case of blunt abdominal trauma. J Radiol Case Rep.

[10] Bennett GL, Balthazar EJ (2003). Ultrasound and CT evaluation of emergent gallbladder pathology. Radiol Clin North Am.

[11] (2024). Radiology Assistant - CT in abdominal trauma. https://radiologyassistant.nl/abdomen/acute-abdomen/ct-in-trauma.

[12] Osada H, Ohno H, Watanabe W, Okada T, Nakada K, Honda N (2010). Cystic artery bleeding due to blunt gallbladder injury: computed tomography findings and treatment with transcatheter arterial embolization. Jpn J Radiol.

